# Drugs or No Drugs?

**Published:** 1887-06-11

**Authors:** 


### Drugs or No Drugs.

"A Consultant," writing in *The British Medical Journal*, says: "In England a certain scepticism has for years been growing up as regards the value of medicinal agents, and has by this time infected a large number of our younger practitioners. . . . Men who have cultivated a contempt for drugs in their student days will, in all probability, have failed to make themselves familiar with their effects and uses, with the result that when they are called upon to divest themselves of that freedom of opinion and practice which are the privileges of hospital life, and have to deal with the public on terms of equality, they find their ignorance of the means of gratifying the whims and prejudices of their patients a terrible hindrance to a successful career."

That is a very Johnsonian sentence as regards its length, but the opposite of Johnsonian as regards its morality. Does "A Consultant" mean to imply that the principal use of drugs is to gratify the "whims and prejudices of patients," and by so doing to lead the practitioner to a "successful career"? And does he consider it a justifiable and desirable thing that young practitioners should secure a "successful career" by prescribing drugs with the sole object of gratifying the "whims and prejudices" of patients? If he does not, he has failed to make it clear that he does not; and if he does he has acted wisely in writing under a *sobriquet* which conceals his identity.

Medical men profess to have a very great objection to professional advertising, and it may be fervently hoped they will long continue to feel such an objection; but if an honest man were called upon to make a choice between a doctor who openly advertised himself and his nostrums, and one who, whilst professing to act on the highest principles, deliberately prescribed that which he knew would be costly and yet useless to his patients, merely to gratify their whims and so to secure his own success,—such a man, if he were not a fool, would choose the open quack rather than the secret knave. If drugs be of no use, or even of very little use, then the medical profession is made up of scoundrels, and the greatest scoundrels are the Fellows of the College of Physicians.

This question of "drugs or no drugs" is not, as one might suppose from the light way in which it is discussed, a professional shuttlecock or tennis-ball to be tossed about from player to player for idle amusement. What is fun to the doctors may be anything but fun to the patients and their friends, especially at term day when the chemist sends in his bill. Even as a mere affair of cost, if drugs be of no use the plain duty of the College of Physicians is to give an authoritative pronouncement in the interests of a much-swindled public.

But is it really so? Is he the truly scientific practitioner who shrugs his shoulders and affects a lofty contempt for the pill and the physic-bottle? We venture to assert in the most emphatic manner that he is not. If he be, then let him prove his honesty by ceasing to practise an art in which he has no faith. That is a test most easy of application, and one which will conclusively demonstrate the genuineness of his own convictions, however it may affect the scientific position of the question at issue.

If it be asserted that the public take too much physic, that is an entirely different assertion. The public probably takes at least a hundred times more than it needs, or than can possibly be of the slightest benefit to it. If it be affirmed that many doctors prescribe physic with the sole purpose of gratifying the whims of their patients, and so of keeping their feet turned in the direction of the consulting-room, that also is no doubt certain, and as reprehensible as it is true. But that too is a totally different affirmation. The question at issue is, whether drugs are or are not of real service.

To this question there will be two answers by two opposite classes of persons. The experienced and intelligent physician who has practised his art with a sincere desire to relieve the sufferings of his fellow-men and to restore the disabled to health, and who, the better to do this, has searched in hospital wards, in books, by converse with others, by personal observation, and in every other way possible to him, for knowledge and skill—he will say undoubtedly that many drugs are of the greatest possible service to his patients, and that the skilful prescriber is a true benefactor to mankind. On the other hand, the man who has known little of illness himself, who is deficient in sympathy with his kind, who listens impatiently to the recital of small troubles, who regards men and women not as reasoning, sentient, sorrowing, and suffering fellow-creatures, but as aggregations of organs adapted to the performance of certain physiological functions, capable of a variety of interesting and abnormal manifestations which he designates pathological—such a man will probably tell you with what he considers an honest frankness, that "drugs are of no real service, you know; they please the public and are profitable to the chemist, and—that is all."

This young man—for he is probably young, at any rate in experience—may be a very superior person. He is certainly superior to his vocation, and should by all means leave it. What he should turn to it is not easy to say. There were schools after Plato, whose members might have been appropriately described as "philosophers at large." Our young medical sceptic should become a "philosopher at large," and found a school for the better instruction of his fellow-countrymen.

The truth is that the universal affectaton of scepticism is becoming a little tedious. All the world have not been fools until Agnosticus was born. Very good corn crops were grown before the steam-plough was invented, and probably physicians have done patients good who never heard of the *Bacillus Tuberculosis*. It is emphatically a sign of rawness and shallowness to think that the nineteenth century lives in the sunshine, but that every other century spent its days in the coal-cellar. It is clear that the young man either has not read the literature of the past, or has not had the sense to separate the wheat from the chaff, the kernel from the husk. We counsel him to go to school again, and before he essays to sweep out the ocean of the world's experience with his little Partingtonian mop, let him at least put on a pair of spectacles, and try to take some kind of measure of the operation he is so foolishly endeavouring to perform.

